# Single-cell immune profiling reveals immune responses in oral lichen planus

**DOI:** 10.3389/fimmu.2023.1182732

**Published:** 2023-04-06

**Authors:** Qionghua Li, Fei Wang, Yujie Shi, Liang Zhong, Shumin Duan, Wenjing Kuang, Na Liu, En Luo, Yu Zhou, Lu Jiang, Hongxia Dan, Xiaobo Luo, Dunfang Zhang, Qianming Chen, Xin Zeng, Taiwen Li

**Affiliations:** ^1^ State Key Laboratory of Oral Diseases, National Clinical Research Center for Oral Diseases, Chinese Academy of Medical Sciences Research Unit of Oral Carcinogenesis and Management, West China Hospital of Stomatology, Sichuan University, Chengdu, Sichuan, China; ^2^ Department of Oral and Maxillofacial Surgery, West China Hospital of Stomatology, Sichuan University, Chengdu, Sichuan, China; ^3^ Department of Biotherapy, State Key Laboratory of Biotherapy and Cancer Center, West China Hospital, Sichuan University, Chengdu, Sichuan, China; ^4^ Collaborative Innovation Center for Cancer Personalized Medicine, Nanjing, China

**Keywords:** single-cell transcriptome sequencing, oral lichen planus, immune repertoire, immune responses, immune cells

## Abstract

**Introduction:**

Oral lichen planus (OLP) is a common chronic inflammatory disorder of the oral mucosa with an unclear etiology. Several types of immune cells are involved in the pathogenesis of OLP.

**Methods:**

We used single-cell RNA sequencing and immune repertoire sequencing to characterize the mucosal immune microenvironment of OLP. The presence of tissue-resident memory CD8+ T cells are validated by multiplex immunofluorescence.

**Results:**

We generated a transcriptome atlas from four OLP biopsy samples and their paired peripheral blood mononuclear cells (PBMCs), and compared them with two healthy tissues and three healthy PBMCs samples. Our analysis revealed activated tissue-resident memory CD8+ T cells in OLP tissues. T cell receptor repertoires displayed apperant clonal expansion and preferrential gene pairing in OLP patients. Additionally, obvious BCR clonal expansion was observed in OLP lesions. Plasmacytoid dendritic cells, a subtype that can promote dendritic cell maturation and enhance lymphocyte cytotoxicity, were identified in OLP. Conventional dendritic cells and macrophages are also found to exhibit pro-inflammatory activity in OLP. Cell-cell communication analysis reveals that fibroblasts might promote the recruitment and extravasation of immune cells into connective tissue.

**Discussion:**

Our study provides insights into the immune ecosystem of OLP, serving as a valuable resource for precision diagnosis and therapy of OLP.

## Introduction

Oral lichen planus (OLP) is a chronic inflammatory disease of the oral mucosa that affects approximately 0.89% of the general population (1). It is characterized by white patches, redness, and bilateral lacy striations on the oral mucosa, which can be accompanied by erosive soreness (2). OLP is considered an oral potentially malignant disorder (3), with a reported transformation rate to malignancy of approximately 1.14% based on large-scale systematic review (4).

OLP can be triggered by various factors, such as hypersensitivity responses, psychological stress, viral infection, and autoimmune reaction (5). Although the pathogenesis of OLP is not fully understood, it is thought that antigen presented by keratinocytes in OLP can trigger keratinocytes apoptosis of oral mucosa (6). Previous studies have shown that multiple immune cell types are involved in the progression of OLP. Myeloid dendritic cells (mDCs) and plasmacytoid dendritic cells (pDCs) are involved in activating T cells and releasing cytokines (IL-12, IL-18, TNF-α, IFN-α) and chemokines in the oral mucosa in individuals with oral lichen planus (5). Th1 cells can produce the proinflammatory cytokine IFN-γ to facilitate the inflammation of OLP (7). Th17 cells are associated with erosion of the oral mucosa by generating IL-17 (8). CD8+ T cells have been shown to produce perforin and granzyme, which can kill keratinocytes in the oral mucosa, further contributing to the destruction of the epithelium (5). Natural killer (NK) cells are found in OLP lesions and play a role in destroying living cells and yield TNF-α and IFN-γ (9). B cells have also been commonly identified in OLP lesions (10). Granules and inflammatory mediators discharged by mast cells, along with the matrix metalloproteinases produced by T cells, can damage the basement membrane of the oral mucosa of OLP (11). These studies indicated that immune cells interact in a complex microenvironment and produce cytokines, chemokines, and other inflammatory mediators that contribute to the initiation and progression of OLP. However a comprehensive single-cell characterization of the immune response in OLP has yet to be reported.

In this study, we aimed to gain a deeper understanding of the immune profile and characterize the molecular features of cells and their interactions within the microenvironment of OLP using single-cell transcriptome and immune repertoire sequencing of OLP. By conducting this study, we were able to uncover the unique immunological responses and clonal features of lymphocytes in individuals with OLP. Our findings provide new insights into the complex ecosystem of OLP and have the potential to inform the development of new therapeutic strategies by monitoring the immune system.

## Materials and methods

### Patient recruitment and sample acquisition

This study was approved by the Institutional Review Board of West China Hospital of Stomatology. All participants provided their informed consent before participating in the study. The diagnosis of OLP was made based on the modified World Health Organization diagnostic criteria (12). A total of four pairs of peripheral blood and buccal mucosa biopsy samples were collected from OLP patients prior to any treatment. Additionally, two buccal mucosa samples and three peripheral blood samples were collected from healthy individuals during orthognathic surgery as a comparison group. All clinical characteristics of the participants are outlined in **Supplementary Table 1**.

### Single-cell suspension preparation

The oral mucosa samples collected were immediately processed. The tissue samples were minced and underwent enzymatic digestion using the human Whole Skin Dissociation Kit (Miltenyi Biotec; No. 130-101-540) at 37°C. The digested sample was then filtered through a 70 μm cell strainer (Miltenyi Biotec; No. 130-110-916) to remove large particles. After centrifugation, the supernatant was removed, and the remaining cell deposit was resuspended in Red Blood Cell Lysis Buffer (Abcam; ab204733) for 5 minutes to lyse the erythrocytes. The cell suspension was washed and resuspended in D-PBS (BBI Life Science; E607009-0500), and magnetic cell separation (MicroBeads, Buffers, Columns, Separators: Miltenyi Biotec) was used to sort live cells. Fresh blood samples were collected in EDTA tubes, diluted with D-PBS, and then transferred onto Ficoll-Paque isolation solution (GE Healthcare; 17-5442-02). Peripheral blood mononuclear cells (PBMCs) were slowly and carefully collected after density gradient centrifugation, and then added with Red Blood Cell Lysis Buffer for 5 min. Afterward, PBMCs were washed twice with D-PBS.

### Library construction and sequencing

The scRNA and VDJ libraries were constructed using Chromium Single Cell V(D)J Reagent Kits (v1 Chemistry, 10X Genomics) according to the manufacturer’s protocols. The sequencing of the sample libraries was performed on a Nova-Seq 6000 sequencer (Illumina, USA).

### Single-cell RNA-seq data analysis

The sequencing data from the scRNA and VDJ libraries were processed through the CellRanger v6.1.1 (13) pipeline from 10X Genomics using the GRCh38 genome reference to generate gene expression and T cell receptor (TCR)/B cell receptor (BCR) data. Subsequently, these data were analyzed using the R package Seurat v4.0.3 (14). During the quality control phase, cells that met one of the following criteria were removed: having fewer than 1000 unique molecular identifiers (UMIs), expressing fewer than 300 genes, or having more than 15% transcripts of mitochondrial genes. The ambient mRNA contamination was removed by SoupX package (15). The R package “DoubletFinder” (16) was applied to predict and remove doublets.

### Trajectory inference analysis

To capture the potential trace of differentiation among different immune cell subtypes, the R package Monocle2 (17) was used to inferring pseudotime trajectories of T cell subsets. The cell trajectory was inferred by function “reduceDimension” and “orderCells” with default parameters.

### Differential gene expression and functional enrichment analysis

Differential gene expression analysis was performed using the “FindMarkers” function in the Seurat package with default parameters. This analysis aimed to identify changes in gene expression between groups. The results of the differential gene expression analysis were further subjected to functional enrichment analysis using the clusterProfiler package (18). The gene lists generated from the analysis were evaluated for their enrichment in biological process (BP) terms from the Gene Ontology Resource (19).

### TCR/BCR clonotype analysis

The TCR clonotypes were identified by analyzing the variable, diversity, and joining genes of the TCR alpha-beta paired chains in conjunction with the nucleotide sequences of the complementarity-determining region 3 (CDR3) regions. Similarly, the BCR clonotypes were defined by analyzing the variable, diversity, and joining genes of the paired light and heavy chains of the BCR along with the CDR3 region nucleotide sequences. The scRepertoire package (20) was employed for the analysis of TCR and BCR data. The UpSetR package (21) was utilized to generate the UpSet plot, while the circlize package (22) was used to create a Chord diagram. Additionally, the STARTRAC package (23) was employed to calculate and plot the tissue migration and state transition indices of TCR clonotypes.

### Cell-cell communication analysis

The interaction between different cell types were inferred by CellChat package (24). Function “rankNet” was applied to comparing the overall information flow of signaling pathways.“netVisual_ aggregate” function was used to visually compare signaling pathway networks “netVisual_bubble” function was utilized to comparing communication probabilities mediated by ligand-receptor pairs.

### Multiplex immunofluorescence staining

Multiplex immunofluorescence staining assays were performed to validate the existence of CD8+Trm cells in oral mucosal tissue of OLP patients and healthy controls. The tissue sections were incubated with the primary antibody anti-CD8 (rabbit; Abcam, USA; Cat. no. ab237709;1:1000) for 1h at room temperature and anti-CD69 (rabbit; Abcam, USA; Cat. no. ab233396;1:500) for overnight at 4°C. Then these sections were incubated with secondary antibody Opal Anti-Ms + Rb HRP (Akoya Biosciences,USA; Cat. No. ARH1001EA). Images were collected using Vectra 3 Pathology Imaging System (Akoya Biosciences,USA).

### Statistical analysis

All statistical analyses were conducted using software R (https://www.R-project.org/) v4.2.0. The detailed descriptions of statistical methods are stated in the section of figure captions.

## Results

### Single-cell transcriptional profiling dissects the cellular ecosystem of OLP

**Figure 1:**
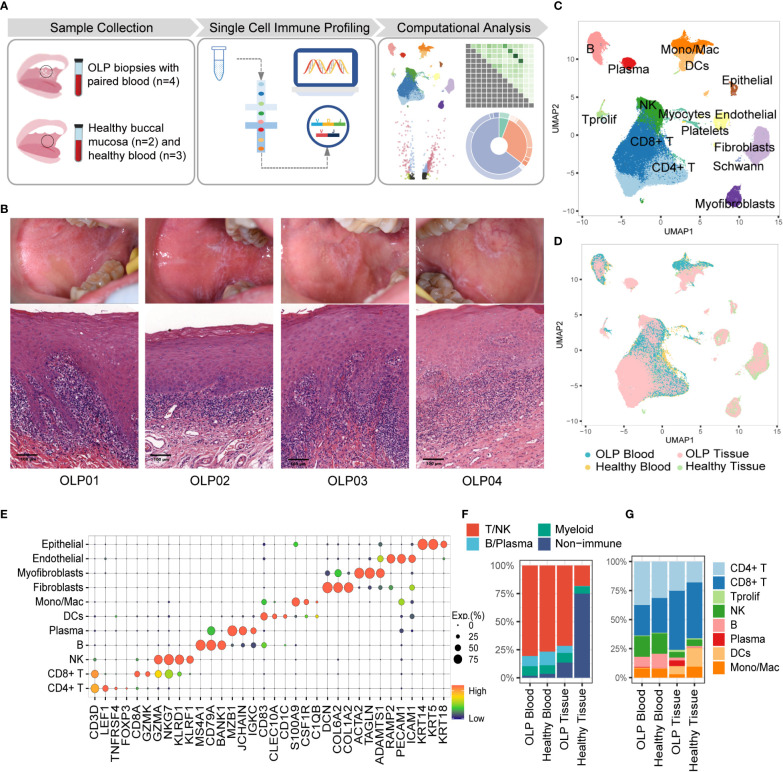
Single-cell landscape of buccal mucosa and blood from OLP patients and healthy controls. **(A)** The work flow of experimental design. **(B)** The clinical manifests and HE staining of OLP lesions. **(C)** The uniform manifold approximation and projection (UMAP) for all cell types, showing cell types by color. CD4+T cells, n=24301; CD8+T cells, n=28673;Tprolif,n=767; NK, n=11094; B cells, n=5889; Plasma cells, n=1694; DCs, n= 3167; Mono/Mac, n=4043; Fibroblasts, n=7163; Myofibroblasts, n=2011; Endothelial cells, n= 2277; Epithelial cells, n=777; Platelets, n=1113; Schwann cells, n=205; Myocytes, n=44. **(D)** The UMAP showing cell origins by color. **(E)** The dot plot showing the expression of marker genes for each cell type. **(F)** The proportion of major immune cells and non-immune cells. **(G)** The percentage of subtypes of immune cells.

Single-cell RNA sequencing and TCR & BCR repertoire sequencing were performed on buccal mucosal biopsy samples and PBMCs from treatment-naïve patients with OLP, as well as location-matched healthy samples and PBMC samples from healthy donors (**Figure 1A**). The histological examination of OLP lesions through Hematoxylin and Eosin (HE) staining revealed the presence of pathological changes, characterized by the infiltration of inflammatory cells in the superficial lamina propria (**Figure 1B**).

To better characterize the cellular heterogeneity, we used Harmony algorithm (25) to correct batch effects for technical variation (**Figure 1D**, **Supplementary Figure 1**) and identified 15 distinct cell types based on marker gene expression (**Figures 1C, E**). The proportions of multiple cell types differ between OLP samples and healthy tissues. An overwhelming majority of cells in OLP tissues are T/NK cells (**Figure 1F**), reflecting the presence of an immune-infiltrated microenvironment in OLP tissues (26). Additionally, B/plasma cells were relatively limited in healthy tissues the proportion of plasma cells in the blood of OLP patients was similar to that in the blood of healthy controls (**Figure 1G**).

### Tissue-resident memory CD8+ T cells are activated in OLP

**Figure 2:**
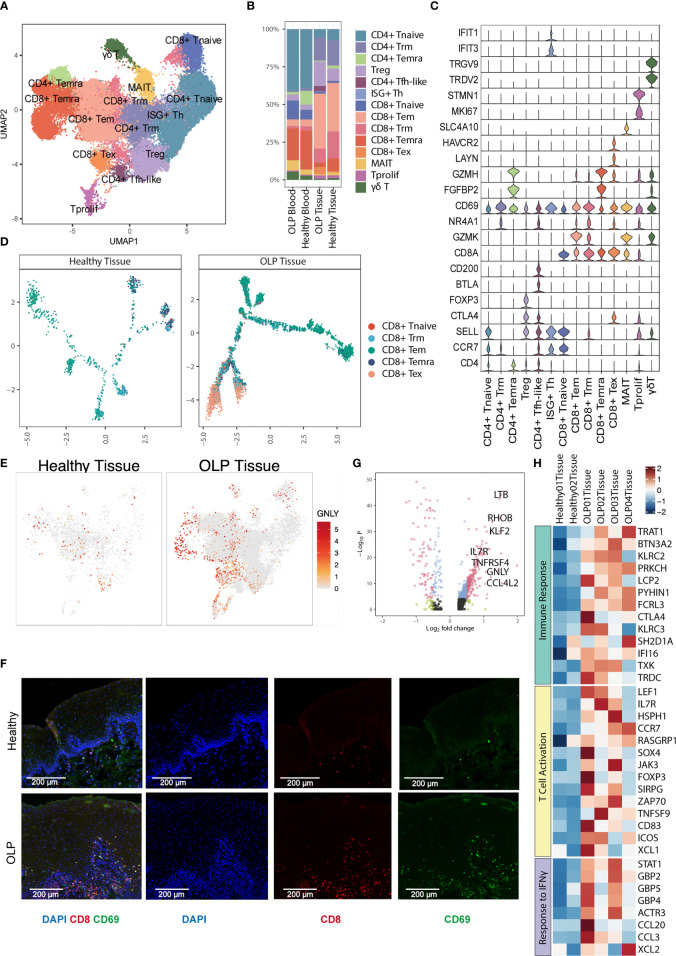
Characterization of T cell subsets. **(A)** The UMAP plot showing 14 T cell subtypes. **(B)** The percentage of T cell subtypes across each group. **(C)** Violin plots showing the expression levels of cell markers (y-axis) in each subtype (x-axis). **(D)** Trajectory analysis for CD8+ T cells between healthy tissue and OLP tissue. **(E)** The UMAP plot showing the expression distribution of GNLY gene. **(F)** The multiplex immunofluorescence staining of CD8 and CD69 for CD8+ Trm cells in OLP and healthy tissues. Images are representative of three biological replicates in each group. **(G)** The volcano plot showing the differentially expressed genes of CD8+ Trm cells between OLP tissues and healthy controls. The genes with |log_2_FC| > 0.5 and adjusted *P* value < 0.0001 were shown in red.Wilcoxon Rank Sum test was used. **(H)** The heatmap showing expression levels of pathway-enriched genes up-regulated in OLP tissues.

To further investigate the transcriptional changes of T cell in OLP, we re-clustered T cells using canonical T cell markers and identified 14 T cell clusters, including six CD4+ T cell subtypes (CD4+ naïve T [CD4+ Tnaive], tissue-resident memory CD4+ T [CD4+ Trm], terminally differentiated effector memory or effector CD4+ T [CD4+ Temra], regulatory T [Treg], interferon-stimulated positive T helper cells[ISG+ Th],and follicular helper-like CD4+ T [CD4+ Tfh-like] cells) five CD8+ T cell subtypes (naïve CD8+ T [CD8+ Tnaive], effector memory CD8+ T [CD8+ Tem], tissue-resident memory CD8+ T [CD8+ Trm], terminally differentiated effector memory or effector CD8+ T [CD8+ Temra] and exhausted CD8+ T [CD8+ Tex] cells) proliferating T cells, mucosal-associated invariant T cells(MAIT), and γδ T cells (**Figures 2A, C**). Major T cell subtypes in tissue samples included CD8+Tem cells, Treg cells, CD4+ Trm cells and CD8+ Trm cells. (**Figure 2B**).

Subsequently, we investigated the trajectory of CD8+ T cells within the tissues and discovered that in OLP lesions, most of T cells differentiated along the Tem and Tex branches (**Figure 2D**),indicating that T cells may be over activated in OLP lesions. Furthermore, T cells present in OLP tissues exhibited increased cytotoxicity, as evidenced by higher expression levels of cytotoxic gene *GNLY* in multiple cell types (**Figure 2E**).

We used immunofluorescence staining to evaluate the expression of CD8 and CD69 markers and observed a significant prevalence of CD8+ Trm cells in OLP lesions, which primarily located within the lamina propria and to a lesser extent in the epithelium (**Figure 2F**). We also compared the gene expression of Trm cell subclusters in oral mucosa between OLP and healthy group, and identified various inflammation-related genes upregulated in OLP (**Figure 2G**). These genes, such as CCR7 and *IL7R*, were involved in immune response, T cell activation, response to IFN-γ, indicating an immune-activating condition in oral mucosa of OLP lesions (**Figure 2H**). Similar results were found in CD8+ Tem cells (**Supplementary Figure 2A**). The up-regulated genes of CD8+ Tem cells in OLP were related to the biological process of leukocyte cell-cell adhesion, type I interferon production and T cell activation (**Supplementary Figure 2B**).

### Distinct TCR usage in OLP tissue

**Figure 3:**
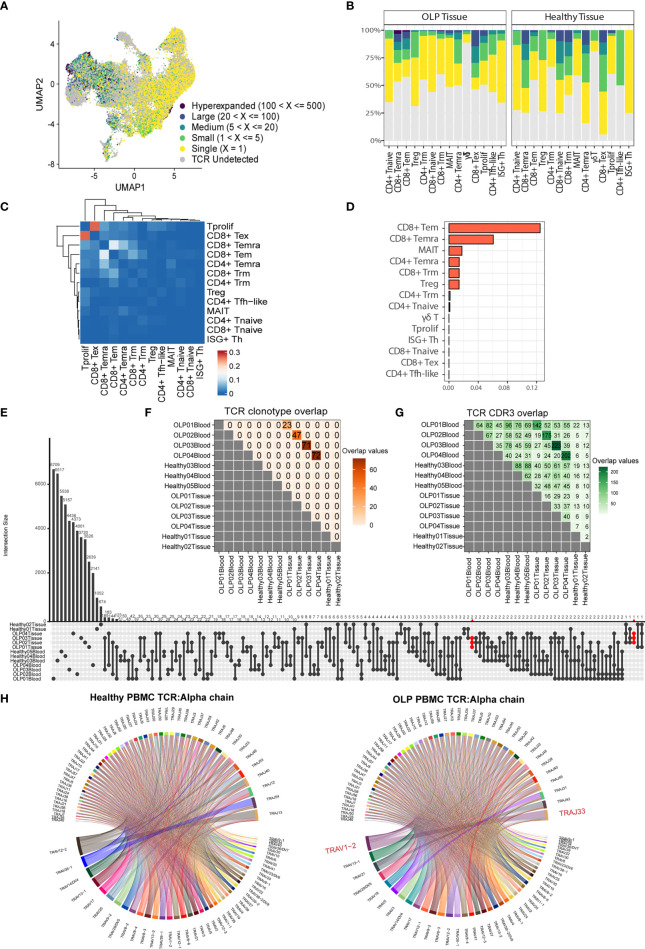
Distinct TCR features in OLP tissue. **(A)** The UMAP plot of T cells colored by the categories of clonal frequency. **(B)** Bar plots showing the distribution of clonal frequency for each subtype, colored by the categories of clonal frequency. **(C)** The transition index among T cell subsets from OLP blood and tissue samples. Red blocks represent a stronger transition process between two cell subsets. **(D)** Ordered migration index among T cell subsets from OLP blood and tissue samples. **(E)** Shared TCR clonotypes among samples. **(F)** Shared TCR CDR3 sequences among samples. **(G)** The upset plot displaying CDR3 sequence sharing across samples. **(H)** Chord diagrams exhibit usage and pairing of V and J genes in alpha chains in OLP blood and healthy blood groups. Links between genes indicate the frequencies of the gene pairs.

To elucidate the clonal characteristics of T cells and the usage of V(D)J genes across different sample groups, we analyzed TCR sequences in combination with scRNA-seq data. An increased frequency of clonotypes was observed in CD8+ T cell clusters, such as CD8+ Tem, CD8+ Temra, and CD8+ Tex subsets (**Figure 3A**). The distribution of T cell clonal expansion varied, with hyperexpanded TCR clones being mainly distributed in CD8+ Tem or CD8+ Temra cells from OLP tissue samples (**Figure 3B**). To investigate the dynamic association between T cells from the oral mucosa and peripheral blood of OLP patients, we used the STARTRAC method (23) to evaluate the migration and transition levels of T cells based on TCR data. Our analysis showed that CD8+ Tem cells exhibited the highest proportion of TCR sharing between peripheral blood and oral mucosa (**Figure 3C**). We also observed frequent clone transitions between CD8+ Tex and proliferating T cells, followed by transitions to CD8+ Temra and CD8+ Tem cells (**Figure 3D**). Based on these results, we inferred that CD8+ Tem cells partially originated from peripheral blood and that some CD8+ Tem cells could transition to CD8+ Tema cells. Moreover, some CD8+ Tex cells were derived from proliferating T cells.

We also investigated the features of clonotypes and CDR3 sequences among individuals or sample groups. Our analysis revealed that while there were some common TCR clonotypes between each OLP patient’s paired samples, there was no TCR clonotype sharing between different OLP patients or between OLP patients and healthy individuals (**Figure 3E**). However, we observed enhanced sharing of CDR3 sequences among samples from different OLP patients compared to healthy samples (**Figure 3F**). Notably, several CDR3 sequences (CAVVSGGYNKLIF, CAVGNTGFQKLVF, CAENNAGNMLTF) were exclusively shared across OLP tissues (**Figure 3G**). Mapping to public VDJ sequences to the VDJdb database (27) revealed a close relationship to the antigen recognition of cytomegalovirus(CMV). Our analysis also revealed that the *TRAV1-2/TRAJ33* pair of alpha chain was preferentially used in TCRs from OLP peripheral blood, and the preferred V/J gene usage pattern of both chains in the oral mucosa of OLP patients was distinct from that in healthy controls (**Figure 3H**; **Supplementary Figures 3A–C**).

### B Cell expansion in OLP

**Figure 4:**
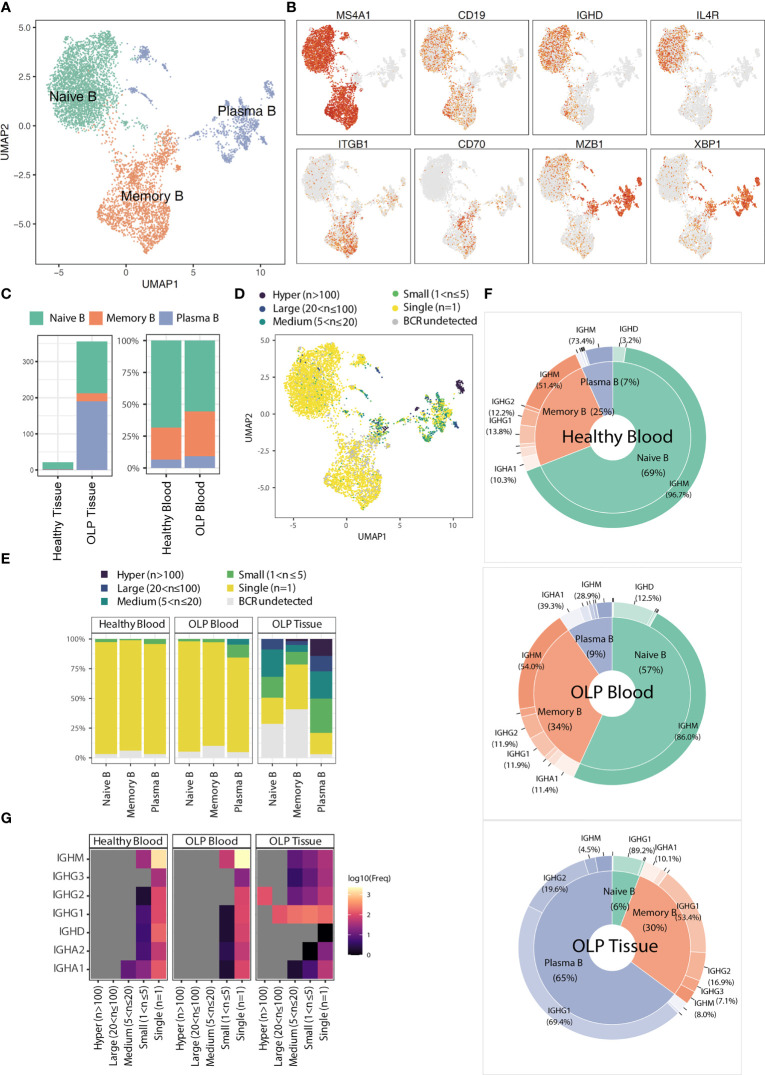
The characteristics of B cell subsets. **(A)** The UMAP plot showing B cell subtypes. **(B)** The UMAP plots showing the expression levels for marker genes. **(C)** The average cell counts of B cell subsets for tissue groups and the proportion of B cell subsets for blood groups. **(D)** The UMAP plot showing the clonotype frequency groups. **(E)** Bar plots showing the distribution of clonal frequency for each B cell subtype across origin groups. **(F)** Radar plots showing the distribution of immunoglobulin classes for each origin group. **(G)** The heatmap showing the frequencies of immunoglobulin types in BCR clonal frequency and origin groups.

Previous studies mentioned the common occurrence of B cells in OLP lesions (10). Therefore, we extracted B cells and identified three common subsets, including naïve B, memory B, and plasma cells, based on typical gene markers (*TCL1A*, *IGHD* for naïve B; *TNFRSF13B*, *CD27* for memory B; *XBP1*, *MZB1* for plasma B) (**Figures 4A, B**). Notably, most of B cells in OLP tissues are plasma cells, while B cells are extremely deficient in healthy tissues (**Figure 4C**).

BCR analysis revealed that the expanded clones were predominantly found in plasma B cells (**Figure 4D**). The BCR clone distributionsbetween OLP blood and healthy blood did not differ significantly. In contrast, a wide range of B cell clones was detected across various B cell subtypes in OLP tissue (**Figure 4E**). In OLP tissue, the predominant immunoglobulin type in both memory B cells and plasma cells was IGHG1, whereas in blood samples, the predominant type was IGHM (**Figures 4F, G**).

### Heightened pro-inflammatory activity of myeloid cells and cell-cell interactions in OLP

**Figure 5:**
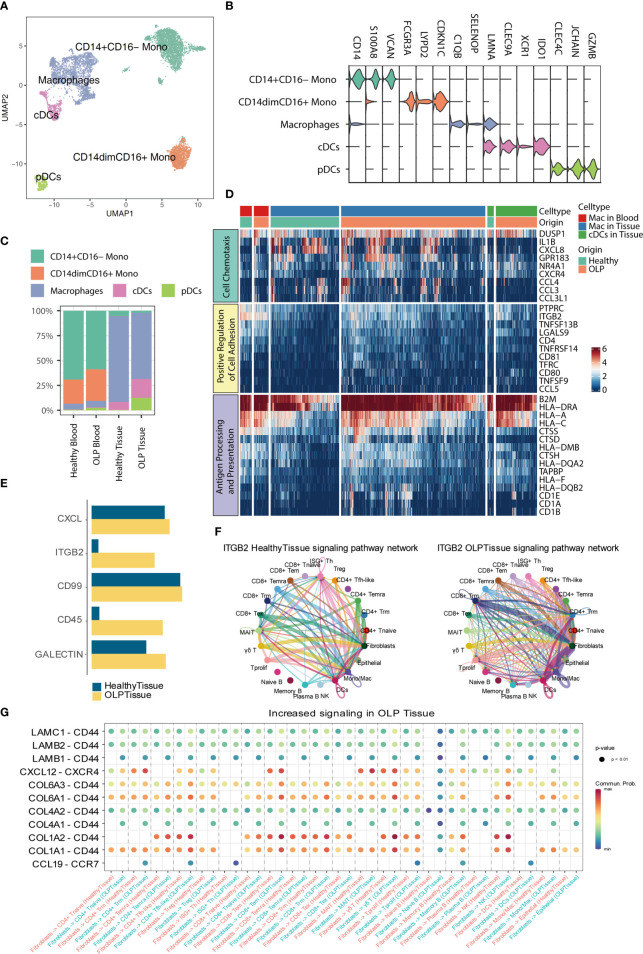
Hallmarks of myeloid cells and cell-cell interaction. **(A)** The UMAP plot showing myeloid cell subtypes. **(B)** The violin plot showing the expression levels of marker genes for each subtype. The expression level of each gene and cell clusters are indicated at the x- and y-axis, respectively. **(C)** Bar plots showing the proportion of myeloid cell subtypes across origin groups. **(D)** The heatmap showing the gene expression enriched for pathways between OLP and healthy group. **(E)** The bar plot displaying the selected signaling pathways in the comprehensive information flow between OLP tissues and healthy tissues. **(F)** Circle plots show the inferred network of the ITGB2 signaling pathway in the above two groups. Edge width indicates the inferred communication probabilities. **(G)** The bubble plot showing selected upregulated signaling ligand-receptor pairs from fibroblasts to other cell types in OLP tissues compared to healthy controls.

Myeloid cells were sub-clustered as classical monocytes (CD14+CD16-), non-classical monocytes (CD14dimCD16+), macrophages, conventional dendritic cells (cDCs), and pDCs, based on canonical markers (**Figures 5A, B**). As shown in **Figure 5C**, monocytes are mainly found in the blood, with no significant difference in proportion between OLP and healthy individuals. In comparison to healthy tissue, OLP tissues show distinct cell subtypes distribution (**Figure 5C**). Although the percentage of cell subtypes did not differ much between the healthy and OLP groups in blood, we found a stronger cellular chemotaxis of macrophages in OLP blood by differential gene expression analysis and enrichment analysis (**Figure 5D**). Also, both macrophages and cDCs in OLP tissues had a stronger ability to positive regulation of cell adhesion and antigen processing and presentation (**Figure 5D**).

To elucidate intercellular and molecular communication in the oral mucosal immune microenvironment of OLP patients, we used CellChat (24) to explore the cell-cell interaction among different cell types. First, we measured the information flow of signaling pathways between the healthy oral mucosa and OLP oral tissues (**Figure 5E**), and noted that the ITGB2 pathway was dominated in OLP tissues. We further compared the inferred network of ITGB2 pathway between OLP and healthy tissue groups (**Figure 5F**), and found that communications between T cell subtypes, myeloid cells and fibroblasts were increased. Then, we compared the ligand-receptor interactions from fibroblasts to immune cells between the two groups (**Figure 5G**), and we identified chemokine-chemokine receptor pairs such as *CXCL12-CXCR4* and *CCL19-CCR7*, are increased in OLP tissues compared with healthy controls. In addition, we found that some ligand-receptor pairs targeted to *CD44* were increased in OLP tissues compared to the healthy tissues. Furthermore, we also compared the ligand-receptor interactions from immune cells to epithelial cells between the OLP and healthy tissue groups (**Supplementary Figure 4**) and found that *ITGB2-ICAM1*, *IFNG-(IFNGR1+IFNGR2)* and *GZMA-F2RL1* were mostly enriched in OLP tissues compared to healthy tissues.

## Discussion

OLP is a chronic inflammatory disorder mediated by T cells, which can result in damage to the oral mucosa and cause pain in patients (28). While several etiological factors and the role of immune cells in the pathogenesis of OLP have been described in previous studies (5), a detailed dissection of the immune ecosystem in OLP is still lacking. In this study, we provided a comprehensive immune cell landscape of OLP at the single-cell level, which allowed us to reveal the role of immune cells and pivotal factors in the pathogenesis of OLP.

Our study provided a comprehensive analysis of the immune cell landscape in OLP at the single-cell level, which revealed the critical role of T cells in the pathogenesis of OLP. We found that T cells, particularly Trm and Tem cells, were enriched in oral mucosal tissues of OLP patients. Additionally, T cells in OLP lesions exhibited increased cytotoxicity and expressed inflammation-related genes, suggesting an immune-activating condition in the oral mucosa of OLP lesions. Our results also showed that T cell activation, cell adhesion, and lymphocyte migration-related pathways were activated in T cells from the oral mucosa of OLP patients. Previous studies mentioned that T cell activation and migration may be involved in the pathogenesis of OLP (29), and some adhesion molecules may be implicated in the migration of lymphocytes to the epithelium in OLP lesions (30). These finding suggested that T cell activation, cell adhesion, and lymphocyte migration may contribute to pathological changes in OLP.

Tissue-resident memory T cells are memory T cells that persist long term in epithelial barrier tissues, such as oral mucosa. Although Trm cells likely evolved to provide rapid immune protection against pathogens, dysregulation of these cells are associated with human inflammatory and autoimmune diseases (31). In our study, we observed an activation of Trm cells in the oral mucosa of OLP patients compared to healthy controls. These transcriptional changes in mucosal Trm cells may indicate a destruction of mucosal immune homeostasis in OLP.

Previous studies have identified stress and viral infections as potential triggers for OLP (32). Our study found that T cells in the oral mucosa of OLP patients displayed signs of inflammation related to viral infection, with some public CDR3 sequences highly relevant to CMV. Biased usage of the *TRAV1-2/TRAJ33* pair was observed in OLP blood samples. MAIT cells are characterized by semi-invariant TCRs (*TRAV1-2/TRAJ 33/12/20*) in humans (33). It can be inferred that this type of preferential pairing in OLP PBMCs mainly originates from MAIT cells. Some studies suggested that MAIT cells may have a role in the onset and chronicity of OLP (34). The public CDR3 sequences and discriminative utilization of V/J genes in OLP patients suggests that some epitopes may trigger the T cell responses under OLP condition.

A distinctive characteristic of OLP is band-like lymphocytic infiltration. Prior research has identified the presence of B cells in the inflammatory infiltrate of all OLP cases, with occasional plasma cells (10). Our investigation revealed the presence of B cells and clonal expansion of B cell receptorss in OLP tissue. Furthermore, IgG1 was found to be the primary antibody in OLP tissue, which has been implicated in pro-inflammatory responses of mucosa (35). These findings suggest that B cells may play a role in the pathological process of OLP.

pDCs have been implicated in exacerbating inflammatory and autoimmune skin diseases, like lupus, as well as oral lichen planus (36, 37). In our study, we observed that pDCs, which are a specific subtype in OLP, have the potential to promote DC maturation and enhance the cytotoxicity of lymphocytes. Additionally, cDCs and macrophages also exhibited pro-inflammatory activity, thus contributing to the infiltration of lymphocytes.

By cell-cell communication analysis, we noted that ITGB2 pathway were highly enriched in OLP tissues, and elevation of ITGB2 signaling were mainly contributed by T cells, myeloid cells and fibroblasts. Previous studies have shown that integrin LFA-1 (encoded by *ITGB2*) plays a critical role in T cell migration (38), adhesion and extravasation (39), indicating that ITGB2 pathway may be closely relate to lymphocyte infiltration in lamina propria of OLP tissue. In addition, we compared the interactions from fibroblasts to immune cells, and interactions from immune cells to epithelial cells, we found that some pro-inflammatory ligand-receptor pairs as well as some ligand-receptor pairs targeted to *CD44* were increased in OLP group. *CD44* has been known to participate in the extravasation of lymphocytes to inflammatory areas (40). Earlier studies also observed increased expression of *CD44* (41), LFA-1 (encoded by *ITGB2*) (42) in OLP lesions compared with healthy oral mucosa by immunohistochemistry. A recent study reported that IFN-γ could enhance the motility and cytotoxicity of CD8 T cells (43). Taken together,these results suggested that fibroblasts might facilitate the recruitment and extravasation of immune cells into connective tissue, some signaling pathways and ligand-receptor pairs may promote the migration of immune cells to the peri-epithelial area and the killing of epithelial cells.

In summary, our study used single-cell transcriptomes and immune repertoires to analyzing the specific ecosystem of OLPand identifying immune responses and molecular changes associated with OLP. Our findings provide new insights into OLP diagnostic biomarkers and potential interventions for OLP.

## Data availability statement

The datasets presented in this study can be found in online repositories. The names of the repository/repositories and accession number(s) can be found below: HRA002370 (GSA).

## Ethics statement

The studies involving human participants were reviewed and approved by Institutional Review Board of West China Hospital of Stomatology. The patients/participants provided their written informed consent to participate in this study.

## Author contributions

QL, FW contributed to acquisition, analysis, and interpretation, drafted and critically revised manuscript. YS, LZ, SD, WK, NL, EL, YZ, LJ, HD, XL, DZ, QC contributed to interpretation, critically revised manuscript; XZ contributed to conception, design, acquisition, and interpretation, critically revised manuscript. TL contributed to conception, design, acquisition, analysis and interpretation, critically revised manuscript. All authors gave their final approval and agree to be accountable for all aspects of the work.
