# Supplementary material for: Single-cell immune profiling reveals immune responses in oral lichen planus
Source: Front Immunol. 2023 Apr 6;14:1182732. doi: 10.3389/fimmu.2023.1182732 (PMC10116058; doi:10.3389/fimmu.2023.1182732)
Supplement: Supplementary file 1 [file DataSheet_1.docx]

Supplementary Material

# Supplementary Figures and Tables

**Supplementary Figures**


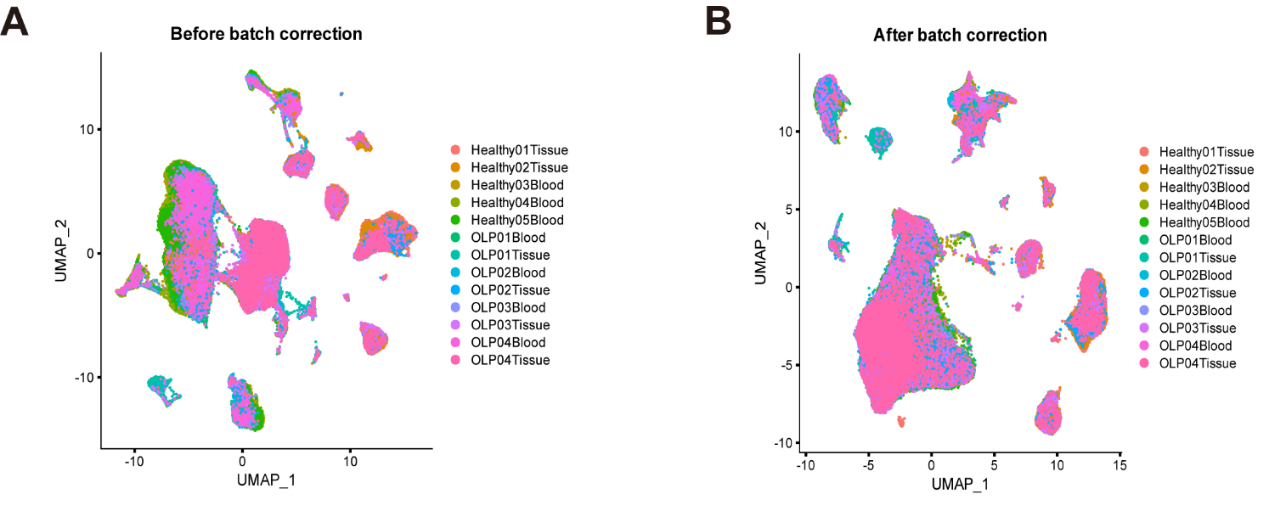


**Supplementary Figure 1.** Effects of batch correction by Harmony. Number of cells from each sample that passed quality control: Healthy01Tissue, 7758cells; Healthy02Tissue, 4314cells; Healthy03Blood, 10736cells; Healthy04Blood, 6348cells; Healthy05Blood, 9950cells; OLP01Tissue, 5932cells; OLP02Tissue, 7012cells; OLP03Tissue, 6853cells; OLP04Tissue, 7246cells; OLP01Blood, 7599cells; OLP02Blood, 7295cells; OLP03Blood, 6374cells; OLP04Blood, 5801cells.

(A) UMAP plot of sample origins before batch correction by Harmony.

(B) UMAP plot of sample origins after batch correction by Harmony.


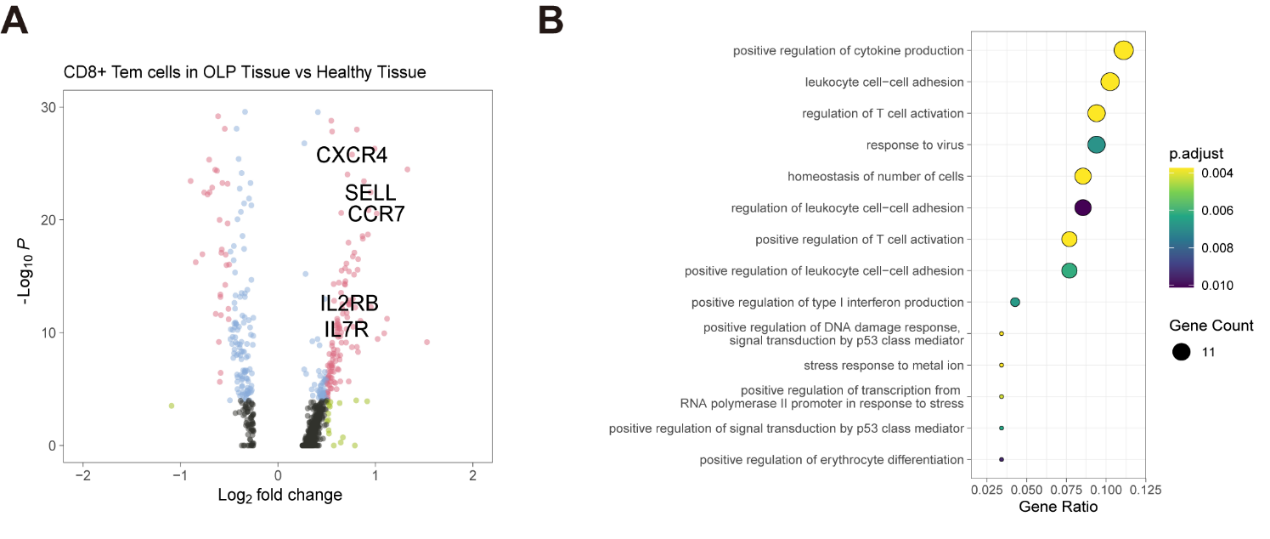


**Supplementary Figure 2.** Differential gene expression analysis and enrichment analysis of CD8+ Tem cells in tissue.

(A) Volcano plot showing the differential expression genes of CD8+ Tem cells in OLP tissues in comparison with healthy controls. Wilcoxon Rank Sum test was used.

(B) Dotplot showing gene enrichment analysis of up-regulated differential expression genes of CD8+ Tem cells in OLP tissues in comparison with healthy controls.


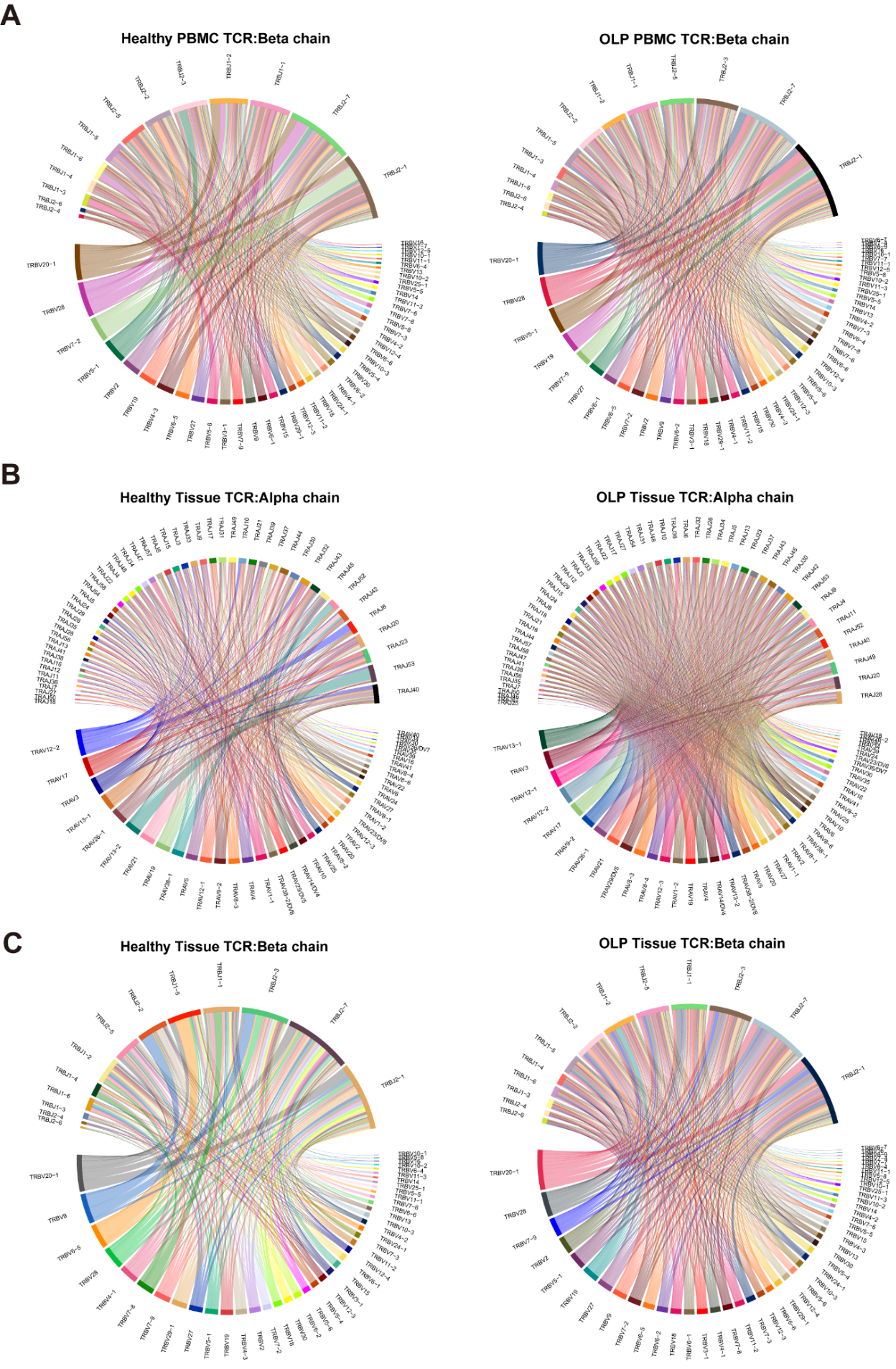


**Supplementary Figure 3.** Usage and paring of V and J genes in TCR chains in different samples.

(A) Chord diagrams exhibits usage and paring of V and J genes in TCR beta chains in healthy blood and OLP blood groups. Links between genes indicate the frequencies of the gene pairs.

(B) Chord diagrams exhibits usage and paring of V and J genes in TCR alpha chains in healthy tissue and OLP tissue groups.

(C) Chord diagrams exhibits usage and paring of V and J genes in TCR beta chains in healthy tissue and OLP tissue groups.


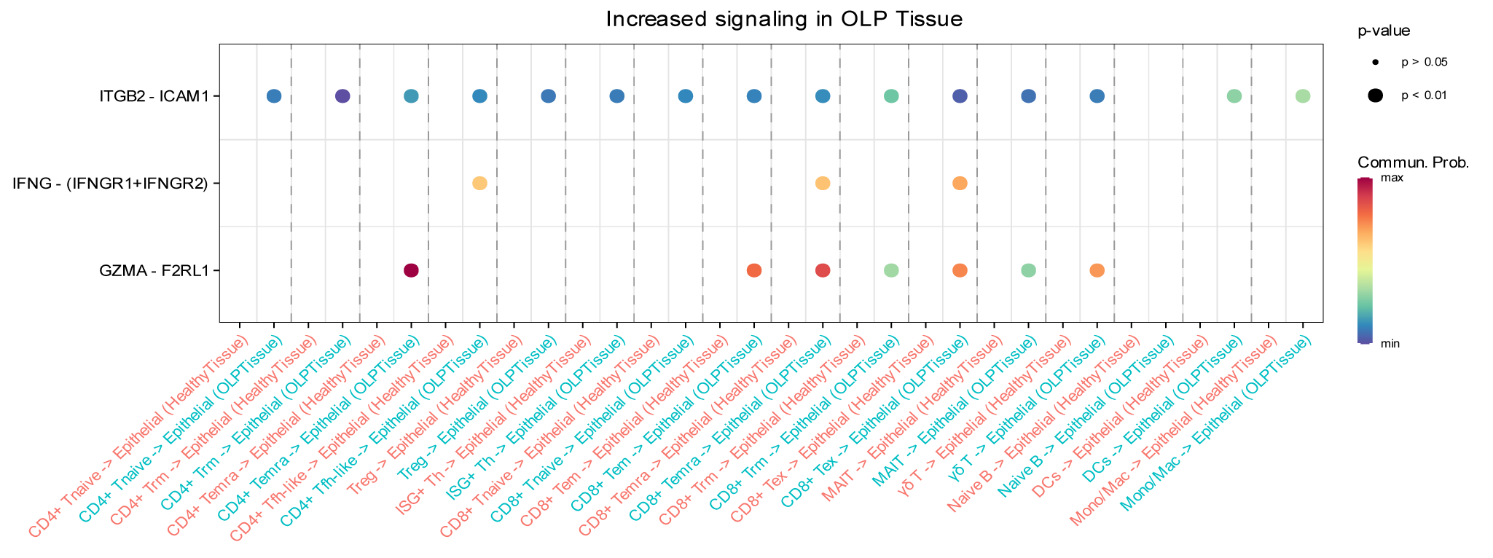


**Supplementary Figure 4.** Bubble plot shows selected increased signaling ligand-receptor pairs from immune cells to epithelial cells in OLP tissues compared to the healthy controls.

**Supplementary Table 1**

Clinical features of enrolled individuals.

| **Patient** | **Age(years)** | **Gender** | **Clinical classification** | **Sample types** |
| --- | --- | --- | --- | --- |
| OLP01 | 23 | Female | Non-erosive | Buccal mucosa;Blood |
| OLP02 | 36 | Male | Non-erosive | Buccal mucosa;Blood |
| OLP03 | 45 | Male | Non-erosive | Buccal mucosa;Blood |
| OLP04 | 44 | Female | Non-erosive | Buccal mucosa;Blood |
| Healthy01 | 24 | Male | Healthy mucosa | Buccal mucosa |
| Healthy02 | 21 | Male | Healthy mucosa | Buccal mucosa |
| Healthy03 | 50 | Male | Healthy blood | Blood |
| Healthy04 | 56 | Male | Healthy blood | Blood |
| Healthy05 | 47 | Female | Healthy blood | Blood |
